# Health system evaluation in conflict-affected countries: a scoping review of approaches and methods

**DOI:** 10.1186/s13031-023-00526-9

**Published:** 2023-06-19

**Authors:** Manar Marzouk, Anna Durrance-Bagale, Sze Tung Lam, Michiko Nagashima-Hayashi, Mengieng Ung, Zeenathnisa Mougammadou Aribou, Ayshath Zaseela, Nafeesah Mohamed Ibrahim, Sunanda Agarwal, Maryam Omar, Sanjida Newaz, Hala Mkhallalati, Natasha Howard

**Affiliations:** 1 Saw Swee Hock School of Public Health, National University of Singapore and National University Health System, 12 Science Drive 2, Singapore, 117549 Singapore; 2 London School of Hygiene and Tropical Medicine, 15-17 Tavistock Place, London, WC1H 9SH UK; 3 Distinguished Careers Institute, Stanford University, Stanford, CA USA; 4 Chelsea and Westminster Hospital NHS Foundation Trust, Fulham Road, London, SW10 9NH UK; 5 Department of Community Health Sciences, Rady Faculty of Health Sciences, University of Manitoba, 750 Bannatyne Ave, Winnipeg, MB R3E 0W2 Canada; 6 Research for Health System Strengthening in North-West of Syria, King’s College London, Strand, London, WC2R 2LS UK

**Keywords:** Health system evaluation, Assessment, Research methods, Conflict

## Abstract

**Introduction:**

Strengthening health systems in conflict-affected settings has become increasingly professionalised. However, evaluation remains challenging and often insufficiently documented in the literature. Many, particularly small-scale health system evaluations, are conducted by government bodies or non-governmental organisations (NGO) with limited capacity to publish their experiences. It is essential to identify the existing literature and main findings as a baseline for future efforts to evaluate the capacity and resilience of conflict-affected health systems. We thus aimed to synthesise the scope of methodological approaches and methods used in the peer-reviewed literature on health system evaluation in conflict-affected settings.

**Methods:**

We conducted a scoping review using Arksey and O’Malley’s method and synthesised findings using the WHO health system ‘building blocks’ framework.

**Results:**

We included 58 eligible sources of 2,355 screened, which included examination of health systems or components in 26 conflict-affected countries, primarily South Sudan and Afghanistan (7 sources each), Democratic Republic of the Congo (6), and Palestine (5). Most sources (86%) were led by foreign academic institutes and international donors and focused on health services delivery (78%), with qualitative designs predominating (53%). Theoretical or conceptual grounding was extremely limited and study designs were not generally complex, as many sources (43%) were NGO project evaluations for international donors and relied on simple and lower-cost methods. Sources were also limited in terms of geography (e.g., limited coverage of the Americas region), by component (e.g., preferences for specific components such as service delivery), gendered (e.g., limited participation of women), and colonised (e.g., limited authorship and research leadership from affected countries).

**Conclusion:**

The evaluation literature in conflict-affected settings remains limited in scope and content, favouring simplified study designs and methods, and including those components and projects implemented or funded internationally. Many identified challenges and limitations (e.g., limited innovation/contextualisation, poor engagement with local actors, gender and language biases) could be mitigated with more rigorous and systematic evaluation approaches.

## Introduction

Evaluating health systems and system components in conflict-affected settings is challenging, yet critical to capture the direct and indirect consequences of violence, insecurity, and service disruptions for affected health systems and populations [66]. Technical support for health systems in conflict-affected settings has become increasingly professionalised since initiation of the humanitarian cluster approach in 2005, including evaluation expectations [24]. However, efforts remain challenging and often insufficiently documented in the healthcare evaluation literature. The practical and ethical challenges involved in implementing research in these settings—constrained access to populations of concern, potential vulnerabilities, building trust, risks for researcher and participant integrity, and problematisation of local ethics approvals when governments are targeting civilians—can discourage robust research efforts [21, 23].

Here we defined ‘conflict-affected country’ as any experiencing “more than 1000 battle-related deaths over a ten-year period, or more than 200 battle-related deaths in any three-year period,” as measured by the Uppsala Conflict Data Program [57]. We used the World Health Organization (WHO) definition of ‘health system’ as “all organisations, people and actions whose primary intent is to promote, restore or maintain health, including efforts to influence determinants of health and more direct health-improving activities” [64]. We also used the WHO definition of ‘health system evaluation’ as “critical assessment, through rigorous processes, of the whole or an aspect of the health system (e.g., governance, financing, workforce, medical products, information, service delivery) to assess whether it fulfils its objectives” [64].

While evaluation to inform the rebuilding of health systems in conflict-affected settings has gained academic traction, less attention has focused on assessing health systems or their components during conflict or on developing conflict-sensitive and contextually appropriate evaluation frameworks to assess systems and programmes in conflict-affected countries. Health systems are complex and often require significant financing to sustain service delivery, while expectations of healthcare among health-workers and service-users may initially be high in settings such as Syria, Iraq, or Ukraine, where pre-existing health services had been comprehensive [22]. This can challenge international and local humanitarian responses during conflict and recovery [40].

Literature assessing the performance of health systems in conflict-affected countries is thus important but limited, partly due to challenges of working and conducting research in these settings but also because assessment is not prioritised during conflict [50]. Both government and humanitarian organisations are often focused on essential services delivery and task-shifting to ensure acute needs are met, rather than evaluating performance or engaging communities [13]. Health systems are often highly fragmented and relatively neglected in conflict-affected countries, with delivery of essential humanitarian aid prioritised and evidence lacking in these settings on how best to evaluate the health system or its components at national and sub-national levels [13, 50].

This review thus aimed to synthesise the scope of methodological approaches and methods used in the peer-reviewed literature on health system evaluation in conflict-affected settings to inform research and debate on the topic. Objectives were to: (i) summarise the scope of existing literature; (ii) synthesise relevant findings; and (iii) consider potential lessons for future evaluations.

## Methods

### Study design

We conducted a scoping review using Arksey and O’Malley’s approach with refinements from Levac et al. and Khalil et al. [32, 34]. We chose a scoping method given the broadness of our topic and anticipated heterogeneity of the literature [7].

### Patient and public involvement

Development of research questions and thematic outcomes was informed by the priorities and experiences of humanitarian practitioners with input from early-career co-authors from lower-income countries. This study was designed to be a standard scoping literature review. Patient involvement was not directly relevant to the conduct of this study as this review drew from publicly accessible data sources.

### Defining the research question

Our research question was: “What is the scope (i.e., extent, nature, distribution) and main findings from the peer-reviewed literature on approaches and methods of health system evaluation in conflict-affected countries?”

### Identifying sources

We searched published literature in five databases systematically (i.e., Embase, Global Health, Medline, Scopus, Web of Science) using MeSH terms and related terminology for ‘conflict-affected countries’ AND ‘health system’ AND ‘evaluation’ adapted to the subject headings for each database. Table 1 provides an example for Medline.

**Table 1:** Search syntax and keywords for Medline

Key word	Medline
Conflict-affected countries	1. MeSH terms ("warfare and armed conflicts"/ or exp armed conflicts/) 2. Conflict* adj3 (affected or zone or zones or Armed or Political or Violent)).mp 3. (War or Wars or Warfare or Rebel* or revolution* or Uprising* or Insurgen* or Complex emergenc*).mp 4. (Political instability or Political unrest).mp 5. 1 or 2 or 3 or 4
Health system	1. MeSH terms ("Delivery of Health Care"/) 1. Public Health Systems Research/ 2. (system* adj3 (care* or health* or healthcare*)).mp 3. 6 or 7 or 8
Evaluation	1. MeSH terms (Program Evaluation/ or Evaluation Study/) 1. MeSH terms Health Impact Assessment/ 2. MeSH terms Process Assessment, Health Care/ 3. (health* adj3 (process* or assess* or effect* or impact* or sustain* or qualit* or measure* or evaluat*)).mp 4. 10 or 11 or 12 or 13 5. 5 and 9 and 14

The * represents a wildcard to facilitate searching of the literature which contains a certain prefix and their variations

### Selecting sources

Table 2 provides our eligibility criteria. Outcomes were restricted to descriptions of evaluation approaches or methods implemented. Source types were restricted to academic and technical literature. Document language was not restricted if an English abstract was available. All study designs, interventions, and participants (e.g., health-workers, expert panels, service-users) were considered. First, we removed duplicates using the reference manager Mendeley. Second, we screened titles and abstracts against eligibility criteria to remove irrelevant documents using Rayyan software [47]. Third, we screened remaining full texts against eligibility criteria to remove ineligible documents and obtain our total number of sources. For potential sources we could not access as full texts, we requested copies from corresponding authors via email. Those we still could not access were screened as abstracts against eligibility criteria and included if they provided useful primary data, such as insight into evaluation frameworks or methods.

**Table 2:** Eligibility criteria

Criteria	Inclusion	Exclusion
Context	Includes a conflict-affected setting (eg, peri-conflict, ‘post-conflict’)	Context is not a conflict-affected country, territory, or subnational setting
Topic	Includes a health system evaluation/assessment or health system component evaluation/ assessment (eg, governance, financing, workforce, medical products, information, service delivery)	Does not include any evaluation of the national or subnational health system or its components
Outcomes	Describes an evaluation approach or method/s and results	Does not describe any health system evaluation approach, methods, or results
Source type	Includes primary research findings using any study design and methods	Does not include primary research (eg, commentary, history, literature review only)
Time-period	Publication date and data collected in 2000 or after	Publication date or data collected before 2000
Language	Any language if it includes an English abstract	No English abstract accessible

### Charting (extracting) data

We extracted data to an Excel sheet using the following headings: (i) source identifiers, i.e., publication year, lead author, source type (e.g., article, conference abstract, book, report); (ii) source characteristics, i.e., country, study approach (e.g., qualitative, quantitative, mixed-method), participant characteristics; (iii) findings, i.e., methods, evaluation tool/framework, health system components/outcomes (i.e., service delivery, governance, financing, workforce, medical products, information).

### Collating and reporting results

First, we summarised sources according to extent (i.e., number, publication year, type, data collection period), distribution (i.e., publication language, countries included), and nature (i.e., study approach, methods, evaluation tools/ framework used, participant characteristics and their gender, methodological limitations, outcomes included, research leadership). Second, we synthesised data under WHO health system framework components of service delivery, leadership and governance, health workforce, health information, medical products, and financing [64]. Although, this framework is not ideal for conflict-affected settings, most practitioners are familiar with the ‘building blocks’ framework and most sources based their descriptions on WHO health systems framework terms.

## Results

### Literature scope and synthesised findings

#### Extent

**Fig. 1:**
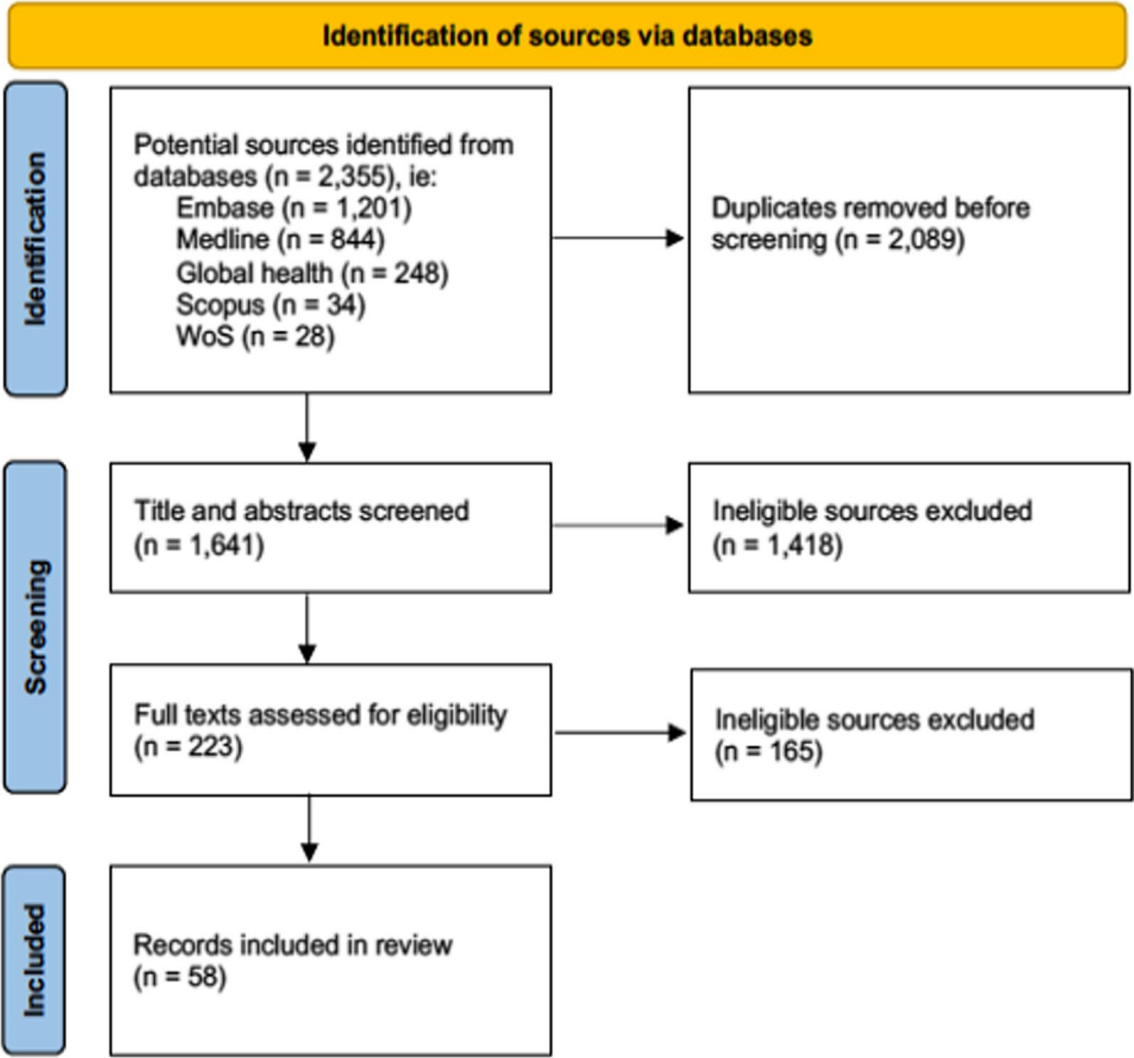
PRISMA flow diagram

**Fig. 2:**
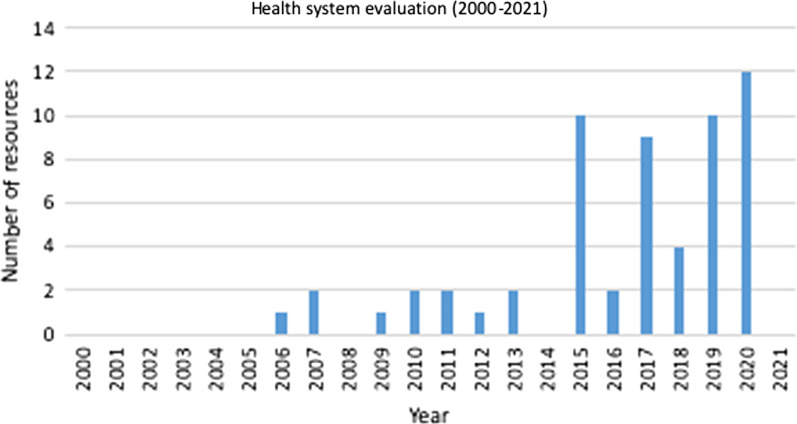
Source distribution by time

We included 58 of 2355 documents identified through database searches (Fig. 1). Most (53) were journal articles, along with 4 abstracts and 1 PhD thesis. Figure 2 shows a gradual increase in sources published, from 1 in 2006 to 12 in 2020 with dips in 2016 and 2018. Data for most sources were collected during conflict, except for 6 in Burundi, DRC, Kosovo, Myanmar, Nigeria, Sri Lanka, and Uganda that reported data collected after conflict [10, 18, 35, 41, 59, 61].

#### Distribution

All sources were published in English. Single-country sources (i.e., 54/58) included 7 (12%) each on Afghanistan and South Sudan, 6 (10%) on the Democratic Republic of the Congo (DRC), 5 (9%) on Palestine; 3 each (5%) on Iraq, Nigeria, and Syria; 2 each (3%) on Myanmar, Sri Lanka, and Ukraine; and 1 each (2%) on Colombia, Congo, Croatia, India, Kosovo, Lebanon, Liberia, Nepal, Pakistan, Sierra Leone, Somalia, Tajikistan, Uganda, and Yemen. Four publications covered two countries each (i.e., 2 on Burundi and Uganda, 1 on DRC and Uganda, 1 on Haiti and South Sudan).

#### Nature

Most sources used qualitative study designs (31/58; 53%), while (16/58; 28%) were quantitative and (11/58; 19%) used mixed methods. Among 31 qualitative sources, most (28/58; 48%) used semi-structured or in-depth interviews, followed by 7 using focus group discussions [9, 17, 25, 36], 1 using storytelling [28], and 1 combining storytelling and ethnography [2]. Of 17 quantitative sources, 12 conducted cross-sectional surveys, 3 (38%) conducted secondary analyses of survey and hospital record data [27, 45, 49], 1 (13%) conducted a cohort study [60], and 1 (13%) conducted an impact evaluation used the TB REACH monitoring and evaluation framework. Of 11 mixed-method approaches, most (7/11) combined semi-structured interview and cross-sectional survey data. For example, Anwari et al. used interviews and surveys to examine a people-centred health systems governance approach in Afghanistan [6].

#### Synthesised findings

We synthesised our findings under three themes: (i) evaluation approaches and challenges; (ii) potential methodological limitations; and (iii) main outcomes by WHO health systems framework component.

### Evaluation approaches and challenges

We identified four issues: (i) minimal holistic health system evaluation; (ii) minimal ‘local’ research leadership; (iii) minimal ‘local’ voices; and (iv) limited data availability and access.

#### Minimal holistic health system evaluation

Only three sources evaluated all health system components, all led by international institutions (i.e., DFID [now FCDO], USAID, LSHTM) to assess health system performance or determine eligibility for additional funding [31, 59, 62]. All used qualitative designs, with semi-structured interviews and focus group discussions the main methods and participants primarily sampled from policymakers at the ministerial level and health-workers employed by international NGOs without mentioning participant gender [31, 59, 62]. For example, Jones et al. interviewed international experts working with international NGOs to explore the feasibility of health system strengthening in South Sudan [31], while Tang and Zhao’s audit of health system performance in northeast Myanmar included community members and local NGOs [59]. All three used WHO’s six health system building blocks to guide analysis, though Warsame et al. added contextual elements such as politics and the peace process in Somalia [62].

#### Minimal ‘local’ research leadership

Only four articles were led by in-country institutions [1, 45, 48, 54]. Ministry of Health authors in Nigeria evaluated the impact of an active case-finding intervention for TB and testing for HIV in displaced communities using the TB REACH monitoring and evaluation framework [1]. Ministry of Health authors in South Sudan assessed health system resilience during protracted conflict using Briguglio’s vulnerability and resilience framework [45]. Universidad de Antioquia authors used the theoretical domains framework (TDF) to identify service-user perceptions about barriers and facilitators to implementing guidelines for the care of amputees in middle-income countries including Colombia [48]. Sengupta et al. conducted a cross-sectional survey to identify health system gaps hindering health literacy promotion and health-seeking behaviours among tribal populations in isolated and conflict-affected Bastar district in India [54].

Most sources (50/58; 86%) were led by foreign institutions or donors, primarily academic (21; 36%) and primarily based in the UK (43%) or US (38%). For example, Nidzvetska et al. evaluated maternal and child health for internally-displaced Ukrainians led by Belgian and US-based academics [44]. Twenty-five (43%) evaluations were led by international donors aiming to audit health system performance. These included a DFID evaluation of health system strengthening in Myanmar [59], a USAID evaluation of health system strengthening in Somalia [62], and a Swiss Red Cross evaluation of dynamics and actors driving fragility in South Sudan and Haiti to improve community-based healthcare and hygiene promotion [25].

#### Minimal local voices

Many sources indicated difficulties accessing national and community actors, especially those conducted remotely [56]. For example, Spiegel et al. justified not interviewing any Ministry of Health or national NGO actors in Yemen as none responded to several contact attempts [56]. Instead, investigators used secondary analysis and triangulation to mitigate this absence of in-country perspectives and several rounds of validation with international participants from different organisations [56]. Similarly, Boris and Melita’s survey of USAID-supported mental health services delivery in Croatia did not include local actors and only a few international participants reflected on local authority perspectives [11]. However, Douedari and Howard were able to focus on local authority perspectives in Northwest Syria despite the constraints of remote participant recruitment and data collection, by snowballing from professional contacts and conducting data collection in Arabic [22].

Only 19 sources (33%) included community perspectives, mainly about patient access to and use of health services. For example, Mosleh et al. interviewed 17 service-users in different Gazan health facilities about health service access barriers [38]. Similarly, Ashour et al. interviewed household heads to assess Palestinian experiences of health services during political turmoil in Gaza [8]. Bernasconi et al. surveyed 189 caregivers of children aged 2–59 months to assess an ICRC quality-of-care initiative in Nigeria [10]. Casey et al. interviewed 23 household members to evaluate implementation of a Save the Children contraceptive intervention in DRC [15].

Most sources (39/58; 67%) focused on the perspectives of healthcare providers and policymakers. For example, Erismann et al.’s assessment of the impact of fragility on health governance and implementation of the Swiss Red Crescent community-based healthcare programme in South Sudan and Haiti only interviewed implementers and donors, acknowledging participant opinion was biased toward interventions [25]. Similarly, Warsame et al. noted the main limitation of their health system evaluation in Somalia was the absence of service-user and civil society voices, which authors attributed to limited access and difficulties obtaining informed consent [62]. Anwari’s evaluation of a people-centred health systems governance approach in Afghanistan noted that community perspectives were not reflected due to geographical distance and insecurity [6].

#### Limited data availability and access

Many sources (13/58) noted data gaps. For example, Mandal et al. found only 353 of 540 reports (65%) for 30 health facilities were filed, of which 105 (30%) contained questionable or missing data [36]. Issues included large discrepancies between what was reported in interviews and what was documented in related reports, which repeated the same numbers (e.g., of ‘patients with STIs’) over six consecutive months [36]. Similarly, Das et al.’s mixed-method study examining the effects of conflict on provision of reproductive, maternal, newborn, child and adolescent health and nutrition services in Pakistan reported a lack of data from insecure Federally Administered Tribal Areas [20].

### Potential methodological limitations

We noted several methodological limitations: (i) limited use of theory and frameworks; (ii) dominance of qualitative approaches and methods; (iii) potential bias (e.g., selection, desirability); (iv) insufficient inclusion of women; and (v) preference for investigator (not participant) language.

#### Limited use of theory and frameworks

Theory-informed research and use of theoretical frameworks to guide research were very limited, with only 10 sources (17%) using any frameworks, primarily to support analysis [58]. These included TB REACH monitoring and evaluation framework [1], TDF [48], and the WHO Situational Analysis to Assess Emergency and Essential Surgical Care tool (WHO, 2007b). None of these frameworks were used for holistic evaluation of health systems, but instead for evaluating one health system component, primarily service delivery (7/10). For example, Jamal et al. used Blanchet’s capacity-oriented resilience framework to interpret health system resilience related to the United Nations Relief and Works Agency (UNRWA) delivery of services to Palestinian refugees in Syria [30]. Similarly, Patino-Lugo et al. used TDF to interpret how cognitive, affective, social, and environmental factors influenced individual behaviour of health-workers or service-users in Colombia [48].

#### Dominance of qualitative approaches and methods

Despite the general lack of theory, over half of sources used qualitative single or multimethod study designs. A good example was Aembe et al.’s qualitative multi-method study, which used ethnography and storytelling to examine how health system governance in DRC was characterised by multi-stakeholder engagement and how this de facto networked governance contributed to state formation in this fragile context [2]. However, reliance on qualitative research for most evaluations, which was generally exploratory in nature and only sampled limited numbers and types of participants, makes it difficult to transfer findings and interpretations more broadly, e.g., to other conflict-affected settings [39].

#### Potential bias

Issues around selection and desirability biases were common. Potential selection bias related primarily to conflict-induced risks, if reaching participants was risky and remote methods deemed difficult. For example, Nic Carthaigh et al. noted that insecurity prevented population-based assessment in Afghanistan, which led to selection bias as patients surveyed already had access to healthcare and findings thus underestimated access barriers [43]. Similarly, Chi et al. only recruited women living within health facility catchments or with weekly access to basic healthcare services through mobile outreach, thus missing the perspectives of potential service-users from disadvantaged and remote areas [17]. Another potential source of selection bias was that quantitative data collection tools such as questionnaires were not originally designed for conflict and not always sufficiently adapted. For example, van der Veen et al.’s survey to explore health-worker integration in mental health services in Kosovo was neither designed for conflict nor adapted in collaboration with local experts and academics [61].

Potential social desirability bias—the tendency to underreport socially undesirable attitudes or behaviors and over-report desirable ones—was a recurrent issue, particularly for evaluations conducted by implementing organisations. For example, Carthaigh et al. noted that service-users and health-workers surveyed in Afghanistan were aware that research was conducted by MSF [43]. Similarly, Casey et al. estimated changes in contraceptive prevalence in DRC following Save the Children’s implementation in these clinics.

### Insufficient inclusion of women

Sex and gender considerations were notably problematic. Most sources (37/58; 64%) did not specify participant sex or disaggregate by gender, while 12 of 37 (32%) included primarily male participants and some only included men. This was particularly noticeable in research with healthcare providers and policymakers. For example, Fardousi et al. only interviewed male health-workers in Syria to examine perspectives on security and improving safety [26]. Collier and Kienzler’s study on provision of noncommunicable disease care in Palestine indicated that male participants predominated except for two women doctors [19]. Saymah et al.’s mixed-method assessment of mental health policy and legislation in Gaza only included male participants from the ministries of health and education [53]. Fardousi et al., for example, justified this absence in terms of the limited numbers of women health-workers in besieged areas and women’s concerns regarding safety and confidentiality [26]. Similarly, Murphy’s evaluation of MSF’s Integrated Diabetic Clinic in DRC indicated all health-workers interviewed were male due to limited numbers of women in the field. However, in interpreting such justifications it is worth noting that Alhaffar et al., using Douedari and Howard’s methods, noted no difficulties recruiting and interviewing women health-workers in opposition-controlled northwest Syria [3, 22].

Eight of nine sources that included women’s voices investigated topics related to reproductive and maternal health. The exception was Nic Carthaigh et al.’s evaluation of Afghan patient experiences in accessing and using general health services [43].

#### Language use in data collection and analysis

Choice of data collection and analysis language appeared problematic or insufficiently considered. Less than a third (17/58; 29%) mentioned translating data collection tools. Similarly, only 17 sources clearly articulated efforts to translate data collection tools and recruit/train interviewers who spoke languages with which participants were most familiar and comfortable. As positive examples, Casey & Tshipamba described developing a survey questionnaire in French and translating it into Congolese Swahili for research in DRC [16], while Nagai et al. used Tamil for data collection in Sri Lanka [41]. Altare et al. collected qualitative data in French or Swahili depending on participant preference, which was positive, but were not able to support the main indigenous languages such as Kituba and Lingala [4]. Most sources relied on non-native languages such as English [51]. Burnham’s assessment of the impact of conflict on health services in Iraq was conducted in English, despite Arabic being Iraq’s official language and most familiar to Iraqis [14]. No sources described analysing data in source languages other than English despite the possibility of losing important nuances as described by Douedari et al. [21]. However, transcript data for Douedari and Howard’s investigation of health system governance in opposition-controlled Syria were analysed in colloquial Syrian Arabic despite this not being noted in methods (author correspondence) so it is possible that others may also have been analysed in source languages though this was not articulated in methods and should have been [22].

### Outcomes by framework component

Most sources (45/58; 78%) evaluated aspects of service delivery, such as coverage and services use, with a notable focus on topics that attracted external funding (e.g., reproductive health, maternal and child health). Only 8 examined general health service delivery (Table 3), most of which were limited in scale and assessed INGO implementation, e.g., Save the Children’s population-level survey of contraceptive prevalence in its North and South Kivu operational areas in DRC [16]. Primary methods used for service delivery evaluation included semi-structured interviews and cross-sectional surveys with only two using observational cohort studies. For example, Todd et al. measured incidence and potential predictors of hepatitis C virus and HIV among internally-displaced people in Kabul using cohort methods [60].

**Table 3:** Sources by lead author, country, and themes

Authors (year)	Country/ies	Participant gender	Methods	Lead Institution	Tool/framework	Themes					
						Service delivery	Governance	Financing	Workforce	Medicine	Information
Abstract (N = 4)											
Ashour [8]	Palestine	Not identified	Mixed	Unclear		X					
Rosenburg et al	Liberia	Not identified	Qualitative	Academic (US)					X		
Taira et al	Sri Lanka	Not identified	Quantitative	UN	WHO EESC	X					
Zangana	Iraq	Not identified	Qualitative	Unclear			X				
Journal Articles (N = 53)											
Abdullahi et al. [1]	Nigeria	Not identified	Quantitative	MoH collaboration	TB REACH	X					
Adams et al. (2020)	Sierra Leone	Mostly men	Qualitative	Academic (UK)		X					
Ager et al. (2015)	Nigeria	Not identified	Qualitative	Donor (bilateral)		X					
Akseer et al. (2019)	Afghanistan	Not identified	Quantitative	Donor (multilateral)		X					
Altare et al. [4]	DRC	Not identified	Mixed	Academic (US)		X					
Ameh et al. [5]	Iraq	Not identified	Mixed	Academic (UK)		X			X		
Anwari et al. [6]	Afghanistan	Not identified	Mixed	Donor (bilateral)			X				
Baingana and Mangen [9]	Uganda	Not identified	Qualitative	INGO		X			X		
Bernasconi et al. [10]	Nigeria	Not identified	Quantitative	Non-academic (Swiss)		X					
Boris et al. (2010)	Croatia	Not identified	Mixed	Donor (bilateral)		X					
Burnham et al. [14]	Iraq	Not identified	Qualitative	Unclear		X					
Nic Carthaigh et al. [43]	Afghanistan	Mostly women	Mixed	INGO		X					
Casey et al. [15]	DRC	Men	Quantitative	INGO		X					
Casey et al. (2017)	DRC	Women	Quantitative	INGO		X					
Chi et al. [17]	Burundi-Uganda	Not identified	Qualitative	Academic (Norway)		X					
Chi et al. [18]	Burundi-Uganda	Women	Qualitative	Academic (Norway)		X					
Collier et al. (2018)	Palestine	Mostly men	Qualitative	Non-academic (UK)		X					
Das et al. [20]	Pakistan	Mostly men	Mixed	Academic (national)		X	X	X	X		
Douedari and Howard [22]	Syria	Not identified	Qualitative	Academic (UK, Syrian led)			X				
Erismann et al. [25]	South Sudan and Haiti	Mostly men	Qualitative	Non-academic (Swiss)			X				
Fardousi et al. [26]	Syria	Men	Qualitative	Academic (UK)					X		
Foster et al. ( 2017)	Thailand, Myanmar	Women	Mixed	Non-academic (US)		X					
Hemat et al. [27]	Afghanistan	Mostly men	Quantitative	INGO		X					
Ho et al. [28]	DRC	Mostly men	Qualitative	INGO		X					
Jamal et al. [30]	Syria	Not identified	Qualitative	Academic (UK, Syrian led)	‘absorptive, adaptive, transformative’ resilience framework	X					
Jones et al. [31]	South Sudan	Not identified	Qualitative	Academic (UK)		X	X	X	X	X	X
Kabakian-Khasholian (2013)	Lebanon	Women	Quantitative	Academic (national)		X					
Kozuki et al. (2018)	South Sudan	Not identified	Mixed	INGO		X					
Malembaka et al. [35]	DRC	Mostly men	Quantitative	Donor (bilateral)		X					
Mandal et al. (2006)	Southern Sudan	Not identified	Qualitative	INGO		X			X	X	
Mosleh et al. (2020a)	Palestine	Mostly men	Qualitative	Non-academic (Swiss)		X					
Mosleh et al. (2020b)	Palestine	Mostly men	Qualitative	Unclear		X					
Murphy et al. (2017)	DRC	Men	Qualitative	INGO	RE-AIM	X					
Nagai et al. [41]	Sri Lanka	Not identified	Mixed	Unclear		X					
Newbrander et al. [42]	Afghanistan	Not identified	Quantitative	Donor (bilateral)				X			
Nidzvetska et al. [44]	Ukraine	Women	Qualitative	Academic (US and Belgium)		X					
Odhiambo et al. [45]	South Sudan	Women	Quantitative	MoH	vulnerability and resilience framework		X				
Patino-Lugo et al. [48]	Colombia	Not identified	Qualitative	Academic (national)	TDF	X	X	X			
Price et al. (2013)	Nepal	Not identified	Quantitative	Academic (US)		X					
Rechel et al. (2010)	Tajikstan	Not identified	Qualitative	Non-academic (EU)			X				
Roberts et al. [51]	Ukraine	Not identified	Quantitative	Academic (UK-Ukraine collaboration)		X					
Sami et al. (2018)	South Sudan	Not identified	Qualitative	Academic (US)	CFIR	X					
Sami et al. (2017)	South Sudan	Women	Quantitative	Academic (US)		X					
Saymah et al. [53]	Palestine	Men	Qualitative	Academic (UK)	WHO-AIMS	X					
Sengupta et al. [54]	India	Not identified	Quantitative	Local NGO		X					X
Spiegel et al. [56]	Yemen	Not identified	Qualitative	Donor (bilateral)	Global Task Force on Cholera Control framework		X				
Taira et al. (2009)	Myanmar	Not identified	Qualitative	Donor (bilateral)		X	X	X	X	X	X
Tang et al. (2019)	Afghanistan	Not identified	Qualitative	Donor (bilateral)		X					
Tappis et al. (2016)	Afghanistan	Not identified	Quantitative	Academic (US, collaboration with MoH)		X					
Valadez et al. (2020)	South Sudan	Not identified	Quantitative	Unclear		X					
van der Veen et al. [61]	Kosovo	Not identified	Mixed	Donor (bilateral)					X		
Warsame et al. [62]	Somalia	Not identified	Qualitative	Donor (bilateral)		X	X	X	X	X	X
Witter et al. [63]	DRC-Uganda	Not identified	Qualitative	Academic (UK-Uganda collaboration)	Adapted strategic purchasing framework			X			
PhD Thesis (N = 1)											
Aembe (2017)	Congo	Not identified	Qualitative	Academic (Netherlands)			X				
Total sources (%)					10 (17)	45 (78)	13 (22)	7 (12)	10 (17)	4 (7)	4 (7)

Thirteen sources (22%) evaluated aspects of health system governance, with a focus on resilience, coordination, fragility, and health system strengthening approaches that were funded by international donors. Almost all these sources focused on the perspectives of central-level officials and partner agencies, including staff at ministries of health, national and international NGOs, and donor organisations, with male voices predominant. For example, Erismann et al.’s investigation of fragility drivers in South Sudan and Haiti primarily included male participants [25].

Ten sources (17%) evaluated aspects of health workforce (Table 3), focusing on health-worker challenges in delivering services in conflict-affected areas. Only three examined health workforce specifically. For example, Fardousi et al. used a qualitative design with remote semi-structured interviews to explore health-worker perspectives on security, improving safety, managing constrained resources, and handling mass casualties during besiegement in Syria [26]. Rosenburg et al. examined factors contributing to community health-worker (CHW) retention in Liberia, similarly using qualitative interviews with CHWs to explore their reasons for becoming CHWs, perspectives on their work, and ways their work impacted their and their families’ lives [52]. Van de Veen et al.’s mixed-method approach examined how staff wellbeing was integrated into the primary healthcare system in Kosovo [61].

Seven sources (12%) included any evaluation of health system financing, with only two focused on financing specifically [42, 63]. In one, USAID aimed to estimate per capita and unit costs for providing a basic package of health services as part of health system rebuilding in Afghanistan, conducting a costing survey of a representative sample of six major NGOs [42]. In the other, a Ugandan academic institute examined effects of Results-Based Financing (RBF) interventions on healthcare purchasing in Uganda and DRC using key informant interviews with international, national, and district-level stakeholders [63].

Six sources (10%) included human resources, but only as a component of evaluation (Table 3). For example, Ameh et al.’s evaluation of challenges to providing Emergency Obstetric Care in Iraqi hospitals [5] and Baingana & Mangen’s evaluation of health-worker capacity in three Ugandan districts as part of an intervention to expand mental health support for war-affected communities led by a Dutch organisation [9].

Only four sources (7%) evaluated medical products, all as a sub-component in tools used to assess other health system components. For example, Mandal et al.’s assessment of IRC health facilities in South Sudan included questions about sexually-transmitted infection drugs and condom availability in the data collection tool [36].

Similarly, only 4 (7%) sources evaluated health information, all as a sub-component in tools to assess other health system components. For example, Sengupta et al.’s identification of gaps in health-seeking behaviour among tribal populations in conflict-affected Indian district included information access via electronic media and newspaper [54].

## Discussion

### Key findings

This review is the first to identify and synthesise the scope and main methodological approaches and limitations of health system evaluation literature for conflict-affected settings. Our interdisciplinary and multinational/multilingual team was able to search a broad range of potential sources. We identified a heterogeneous literature covering 26 countries, primarily South Sudan, Afghanistan, and DRC. The strength of this literature is in the effort to adapt healthcare evaluation approaches and methods to challenging research environments. However, there were several weaknesses and notable gaps in this literature related to theory and conceptualisation, inclusion and representation, and coverage of health systems components that future research should aim to address.

This literature is largely under-theorised in comparison to the general healthcare and complex system evaluation literatures [55]. The 2010 WHO health system blocks framework was the most frequently used, despite its recognised weaknesses and later revision efforts [22]. However, both its familiarity and the absence of significant theoretical or conceptual innovation in evaluating health systems in conflict-affected settings, means its ubiquity is perhaps unsurprising. The ReBUILD consortium, examining health systems rebuilding after conflict/crises in Cambodia, Sierra Leone, Uganda, and Zimbabwe, suggested including the impact of conflict and intersecting inequalities when evaluating health systems, which are essential for developing responsive health systems after conflict [37].

Underrepresentation was geographic (e.g., minimal examination of health systems in the Americas region), component (e.g., limited holistic evaluation and preferences for specific components such as service delivery), gendered (e.g., limited participation of women), and racialised/colonised (e.g., limited authorship and research leadership from affected countries). Interestingly, while UK and US-based authors dominated this literature, minimal research was actually conducted in conflict-affected countries in the Americas region. In addition to only five sources being led by authors affiliated to in-country institutions, many evaluations appeared to be conducted with limited or no engagement with affected institutions or communities. In conflict-affected settings, given data limitations and access challenges, we would argue that the need to co-design evaluations in close collaboration with remaining health service leadership and affected communities, along with inclusion of women’s perspectives, is even greater than in more secure settings if we want assessments to be effective and relevant. Brewster et al. similarly suggest a concordat approach to enhance collaboration between evaluation partners (e.g., academics, local authorities, designers, funders) so all agree core principles to guide evaluation from the beginning [12].

Coverage of health systems, specific components, and intermediate-advanced methodology were limited. Many sources (43%) were INGO project evaluations for international donors, so relied on simpler and lower-cost methods such as semi-structured key informant interviews and cross-sectional household surveys. As purposive or convenience sampling of participants among central-level policymakers and health system managers in English is significantly faster and easier than engaging and building trust with frontline workers or service-users, this focus is understandable but limited. While all perspectives are potentially useful, they will be considerably different. Only 11 sources mixed qualitative and quantitative methods, and more technical study designs (e.g., quasi-experimental, longitudinal cohorts) or theory-driven implementation science approaches were largely absent. Evaluation efforts focused on health service delivery and human resources, while financing, information, and medical products and technologies were neglected. Similarly, maternal and child services access was prioritised, while access for other potentially vulnerable groups (e.g., people with disabilities; older; lesbian, gay, bisexual, and transgender; displaced) was neglected.

### Implications

The peer-reviewed literature on health systems evaluation in conflict-affected settings is generally limited methodologically and topically in terms of its capacity to provide data and learning to enhance health system effectiveness, efficiency, equity, and humanity [29]. As most researchers will initially examine this literature whether or not they then attempt to access any grey literature, these limitations are important. While funders increasingly encourage implementing agencies to conduct evaluations, due to the ad-hoc and short-term nature of most donor funding these often consist of simple online surveys, interviews of ‘beneficiaries,’ or health-worker satisfaction, which do not provide sufficient holistic data for impact and sustainability of such complex systems. As Woodward et al. highlighted, more capacity-building in health systems research is needed [67]. Lamont et al. further suggested, large-scale changes to provide usable national-scale data and lessons may require independently-funded nationally-representative, or subnationally-representative in the case of fragmented countries such as Syria and Yemen, evaluations [33].

Some limitations in this health system evaluation literature are natural and potentially unavoidable results of conflict, such as missing data and physical inaccessibility of relevant participants. However, others could be relatively inexpensively mitigated, such as including indigenous researchers, including women, and conducting research and analyses in appropriate languages. Douedari & Howard additionally emphasised the need for caution in translating some health system terms from English, as, for example, accountability in Arabic could mean investigation and lack of trust, while legitimacy could have religious connotations [22]. Aembe et al. mentioned the importance of analysing both verbal and paralinguistic (e.g., non-verbal) elements of communication, thus highlighting the importance of ‘localism’ of lead researchers who can speak indigenous languages and understand local context and nuance when interpreting and writing up findings [2].

### Limitations

Standard scoping review limitations apply to this study, including reliance on selected databases and search syntax that may miss documents not indexed or syntaxed according to criteria and lack of formal assessment of source methodological quality due to heterogeneity. Scoping literature review methodology does not require source quality appraisal and we did not appraise quality formally as the quality of this body of literature was relatively limited and primarily demonstrated the need for more robust health system evaluation engagement in conflict-affected settings. Second, while we included all publication languages identified by our search syntax, we did not translate our syntax into other languages so may have missed some relevant documents. Third, we intentionally did not include grey (non-academic) literature as we wanted to determine the current scope of the peer-reviewed publications most researchers would initially examine. However, we recognise that many evaluations are never published, particularly those conducted by locally embedded researchers, and future research should examine whether grey sources include more indigenous voices and different evaluation data. Finally, our choice of the WHO health system ‘building blocks’ framework to structure our findings may seem controversial given its known conceptual limitations and we acknowledge that using it made it harder to explore some nuances related to processes and outcomes. However, we chose it for two reasons; first, because several co-authors were new to qualitative analysis and wanted a conceptually manageable framework and second (and similarly) this remains the main framework used by researchers in conflict-affected settings and many publications we included were organised accordingly. Thus, given the WHO framework is descriptive and we only used it as a means of organising our findings for the two reasons mentioned, we do not consider that using it limited our analysis in a meaningful way.

## Conclusions

The relative increase in health system evaluation literature in conflict-affected settings in the past decade is noteworthy. However, it remains under-theorised and generally limited to assessing health system components using survey or qualitative interview methods with participant samples and languages that may be unsuitable, insufficient consideration of global ‘decolonisation’ of representation and inclusion among both participants and researchers, and restricted health system components and topics primarily driven by international funding requirements rather than health system priorities. Many of these challenges and limitations could thus be mitigated by funding more robust and innovative evaluation methods contextualised to conflict-affected settings, meaningful collaboration with national and subnational actors using relevant languages, and decreasing gender biases in sampling.

## Data Availability

Please contact the corresponding author for data requests.

## Abbreviations

NGO: Non-governmental organisation
WHO: World Health Organization
